# Comparative study on alginate/chitosan microcapsules and Montanide ISA 61 as vaccine adjuvants in mice

**DOI:** 10.1371/journal.pone.0298117

**Published:** 2024-04-04

**Authors:** Laice A. Silva, Monique F. Souza, Thaynara P. Carvalho, Clarissa H. Santana, Andressa C. Guedes, Jefferson Bruno S. Oliveira, Pâmela A. de Lima, Paulo Roberto A. Nogueira, Humberto de Mello Brandão, Tatiane A. da Paixão, Renato Lima Santos

**Affiliations:** 1 Departamento de Clínica e Cirurgia Veterinária, Escola de Veterinária, Universidade Federal de Minas Gerais, Belo Horizonte, Minas Gerais, Brazil; 2 Departamento de Patologia Geral, Instituto de Ciências Biológicas, Universidade Federal de Minas Gerais, Belo Horizonte, Minas Gerais, Brazil; 3 Embrapa Gado de Leite, Juiz de Fora, Minas Gerais, Brazil; UFPL, BRAZIL

## Abstract

Selection of adjuvant to be combined with the antigen is an extremely important point for formulating effective vaccines. The aim of this study was to evaluate reactogenicity, levels of IgM, IgG and subclasses (IgG1, IgG2b and IgG3), and protection elicited by vaccine formulations with association of chitosan coated alginate or Montanide ISA 61 with γ-irradiated *Brucella ovis*. The alginate/chitosan biopolymers as well as the Montanide ISA 61 emulsion elicited intense and long-lasting local response, especially when associated with the antigen. However, Montanide ISA 61 induced less intense reactogenicity when compared to alginate/chitosan. Furthermore, γ-irradiated *B*. *ovis* with Montanide ISA 61 induced higher levels of IgG2b an important marker of cellular immune response. In conclusion, Montanide ISA 61 resulted in milder reactogenicity when compared to the alginate/chitosan, while it induced a high IgG2b/IgG1 ratio compatible with a Th1 profile response.

## Introduction

An adjuvant acts to accelerate, prolong or enhance the antigen-specific immune responses. Adjuvants make the recognition of antigens by the immune system more efficient. Many mechanisms of action have been proposed according to the adjuvant used. Initially, antigenic persistence was considered a depot effect by extending the time which antigen is available in the tissue. Currently, this process is also attributed to a controlled release of antigens and direct activation of innate immune cells that induce the release of cytokines and chemokines through specific receptors, thus influencing the adaptive immune response [1, 2].

Development of novel adjuvants aims to improve and enhance the immune responses induced by vaccines, without compromising their safety. There is no universal or ideal adjuvant for all vaccines. The appropriate choice of adjuvant to be combined with various antigens is a key step when formulating effective vaccines [2, 3].

Novel adjuvants may be more effective for triggering an antibody or a cell-mediated immune response. Particularly, Montanide-based adjuvants have demonstrated superior efficacy in a variety of human and veterinary vaccines [4]. These formulations characterized as oil-in-water, water-in-oil emulsions, or water-oil-water were developed for use in therapeutic and prophylactic vaccines [1]. The Montanide ISA series includes a water-in-oil emulsion Montanide ISA 61 (ISA 61). It consists of mineral oil based solutions that incorporate a specific enriched light mineral oil and a highly refined emulsifier obtained from mannitol and purified vegetable-derived oleic acid. Montanide ISA 61 is characterized by stimulating strong lasting cellular immune responses and was evaluated in studies of vaccines against bovine pathogens [5–8].

Biomaterials including alginate and chitosan have many desirable properties as adjuvants including biocompatibility, controlled and sustained release of antigens, and absence of toxicity. Through encapsulation in microspheres, the combination of alginate and chitosan has been increasingly explored in human and veterinary medicine. Chitosan is an abundant biopolymer derived from natural chitin found in the exoskeleton of arthropods, crustaceans, and fungal cell walls. Alginate is a naturally occurring polysaccharide extracted from the cell wall of brown algae [9–12].

In this study, *Brucella ovis* was employed as a model for intracellular bacteria. Brucellosis may affect various host species and may be caused by many *Brucella* species [13]. In general, *Brucella* spp. have a tropism for genital organs causing infertility in animals [14], and many *Brucella* species can infect humans, although *B*. *ovis* is not zoonotic [13]. Importantly, the mouse has been extensively used for experimental infection and vaccinology of brucellosis [15].

The goal of this study was to evaluate reactogenicity at the inoculation sites and induction of immune response by chitosan coated alginate or Montanide ISA 61 used as adjuvants with γ-irradiated *Brucella ovis* formulations.

## Materials and methods

### Bacterial strain and growth conditions

*Brucella ovis*, an intracellular pathogen that can be safely handled with biosafety level 2 practices was used in this study. The reference wild type (WT) *B*. *ovis* strain (ATCC 25840) was grown on tryptic soy agar (TSA) with 1% hemoglobin for 3 days at 37°C with 5% CO_2_. Bacteria were suspended in sterile PBS, and concentrations were estimated in a spectrophotometer at 600 nm (OD600). Bacterial concentration was adjusted for 10^11^ colony forming units (CFU)/mL, which was irradiated with 15 kilogray (kGy), and stored at -80°C. Irradiation was performed at the Centro de Desenvolvimento de Tecnologia Nuclear (CDTN–Belo Horizonte, Brazil). A volume of 100 μL of the suspension containing irradiated bacteria was plated on TSA and cultured at 37°C with 5% CO_2_ for 72 h to confirm inactivation and sterility.

### Encapsulation of γ-irradiated *Brucella ovis* ATCC 25840 in alginate-chitosan

A suspension containing the equivalent concentration of 10^11^ CFU/mL of γ-irradiated *B*. *ovis* was resuspended in 2 mL of 1% alginate solution (Sigma-Aldrich; product number 448869 with 96.1% deacetylation, and 35 cps of viscosity) and dripped with a 33G needle into 10 mL of polymerization solution (0.5 mM CaCl_2_). After dripping, the microcapsules were homogenized for 15 minutes and washed twice in MOPS buffer (3-[Nmorpholino] propanesulfonic). Microcapsules containing 10^10^ CFU/mL of γ-irradiated *B*. *ovis* were homogenized for 30 minutes in a solution of 1% chitosan (pH 4.55, dissolved in 1% acetic acid) and washed once in MOPS buffer solution for 5 minutes. For the preparation of empty alginate-chitosan capsules, the procedure was similar, but without incorporation of bacteria into the alginate.

### Emulsification of γ-irradiated *Brucella ovis* in Montanide ISA 61

A formulation with γ-irradiated *B*. *ovis* was prepared in ISA 61 VG (SEPPIC-Brazil) by adding 400 μL of γ-irradiated bacteria at a concentration equivalent to 10^10^ CFU/mL to 600 μL of Montanide (40/60 v/v), according to the manufacturer’s instructions, and emulsified with a two-way syringe. The antigen and ISA 61 VG were first processed by 20 cycles of low speed emulsification for 8s/cycle, followed by 60 cycles of high speed emulsification for 1s/cycle. A similar procedure was applied to the formulation which consisted only of adjuvant, by replacing the bacterial suspension with 400 μL of sterile PBS, which was added to 600 μL of Montanide. The preparation was considered successful when the drop floated on the surface and maintained its water-oil emulsified state.

### Animal experiments

Animal experiments were approved by the Institutional Ethics Committee on Animal Experimentation of the Universidade Federal de Minas Gerais (CEUA protocol 4/2021). Mice were kept at 22°C in a 12 h light/12 h dark cycle with water and food *ad libitum*. A total of 25 7-week-old female C57BL/6 mice were randomly divided into five groups (n = 5): (i) γ-irradiated *B*. *ovis* in chitosan coated alginate capsules (γ-irradiated *B*. *ovis* + alginate/chitosan); (ii) γ-irradiated *B*. *ovis* emulsified in Montanide ISA 61 (γ-irradiated *B*. *ovis* + Montanide ISA 61); (iii) empty alginate and chitosan microcapsules (alginate/chitosan); (iv) Montanide ISA 61; and (v) non-immunized control group (inoculated with sterile PBS). Mice were subcutaneously inoculated with 100 μL, twice with a two-week interval. Aiming to reduce reactogenicity all formulations were applied at two inoculation sites, i.e. in the subcutaneous tissue of the cervical and lumbar regions. Vaccine dose contained the equivalent to 10^9^ CFU of γ-irradiated *B*. *ovis*. Twenty eight days after the second dose, mice were challenged intraperitoneally (i.p.) with 10^7^ CFU of wild type *B*. *ovis* (ATCC 25840). Two weeks after challenge mice were euthanized with 2% xylazine (30 mg/Kg) and 10% ketamine (300 mg/Kg) by intraperitoneal route. Samples of spleen were obtained for bacteriologic culture and histopathology. Inoculation sites were also sampled for histopathology.

### Evaluation of lesions at the inoculation site of formulations with adjuvants

Mice were monitored daily throughout the experiment to evaluate the inoculation site and behavioral changes related to pain. Skin thickness was measured by caliper and the weight of animals was also evaluated. Lesions including edema (thickness of the skin), erythema, alopecia and fistulas were classified according to the score: 0 –absence (skin thickness: 1.0 to 1.5 mm, erythema, alopecia and fistulas absent); 1 –mild (skin thickness: 2.0 to 3.5 mm, erythema, alopecia and fistulas absent); 2 –moderate (skin thickness:4.0 to 6.5 mm, erythema and alopecia present, and fistulas absent); 3 –severe (skin thickness: 7.0 to 9.5 mm, erythema, alopecia and fistulas).

### Histopathology and immunohistochemistry

Tissue samples from the inoculation site and spleen of mice were fixed by immersion in 10% buffered formalin for 24 hours, processed for paraffin embedding, sectioned in a microtome (4 μm-thick sections), and stained with hematoxylin and eosin. Lesions in the spleen were scored according to the intensity of the inflammatory infiltrate: 0 –absent, 1 –mild, 2—moderate and 3 –severe; and the absence or presence of necrosis: 0 –absent or 1 –present. The combined histopathology score ranging from 0 to 4. Immunohistochemistry for *in situ* detection of *Brucella* sp. antigens was performed as previously described [16]. Histological sections were incubated with 10% of hydrogen peroxide for 1 hour for blocking endogenous peroxidase, rinsed in PBS, and then incubated with skimmed milk (25 μg/mL) for 45 minutes for blocking non-specific labeling. Sections were then incubated with a polyclonal anti-*Brucella* spp. antibody diluted 1:1000 for 1 hour. Sections were rinsed three times in PBS, and incubated with a detection system (EnVision FLEX+; Dako, Carpinteria, CA, USA) for 30 minutes, followed by rising in PBS, and development with a chromogen 3’3- diaminobenzidina (DAB+ Substrate Chromogen; Dako) for 30 seconds, and sections were counterstained with Mayer’s hematoxylin for 35 seconds. Positive controls included tissue sections from mice experimentally infected with *Brucella* sp. [17]. Negative controls had the primary antibody replaced with sterile PBS.

### Evaluation of humoral immune response

Serum samples were collected during the necropsy by cardiac puncture. Titers of IgM, total IgG and its subclasses, IgG1, IgG2b, and IgG3 were determined by Indirect Enzyme Linked Immunosorbent Assay (ELISAi). ELISA plates (Costar, Sigma-Aldrich, USA) were sensitized with 100 μL of sonicate crude total *B*. *ovis* antigen with a protein concentration of 0.25 μg per well for 18 hours at 4°C. After antigen adsorption, the plates were washed twice with PBST 0.05% Tween 20 (Sigma-Aldrich, USA) and blocked with 200 μL of PBS plus 5% bovine serum albumin (BSA) for 1 hour at 37°C. After blocking, the solution from the wells was removed and samples of animal sera were diluted (1:100) in PBS solution with 2.5% BSA, added to wells, and incubated for 1 hour at 37°C. The plates were washed three times with 0.05% PBST and 100 μL of the secondary anti-mice antibody (IgM, IgG, IgG1, IgG2b, and IgG3) conjugated with peroxidase (Sigma-Aldrich, USA) diluted 1: 2.000 in PBS-BSA 2.5% were added to wells. After incubation at 37°C for 1 hour, the plates were again washed three times with the washing solution, and then 100 μL/well of substrate (0.1 M anhydrous citric acid, 0.2 M sodium phosphate, 0.05% OPD and 0.1% H₂O₂) was added. The plates were protected from light for 5 minutes with the developer solution, and the reaction was stopped by the addition of 50 μL of H_2_SO_4_. The resulting absorbance was recorded in an ELISA reader at 492 nm (MR-96A Microplate reader, Mindray, China). All assays were performed in duplicates.

### Statistical analysis

CFU counts and antibody measurement were normalized by logarithmic transformation and submitted to the analysis of variance (ANOVA). Means were compared using the Tukey test. Evaluation of skin thickness, score of macroscopic skin lesions, and histopathological scores were analyzed using the non-parametric Kruskal-Wallis test followed by the Dunn’s Test. All analyses were performed with GraphPad Prism software version 8.0.1 (GraphPad Prism software 8.0.1, Inc, USA). Values were considered statistically different when P value < 0.05.

## Results

### Vaccine formulations with adjuvants elicit an intense and long-lasting inflammatory reaction at the site of inoculation

Mice inoculated with γ-irradiated *B*. *ovis* + alginate/chitosan displayed discomfort during the procedure to measure the thickness of the skin at the inoculation site. That behavior was not observed in mice immunized with γ-irradiated *B*. *ovis* + Montanide ISA 61 or those that received only the adjuvants (alginate/chitosan and Montanide ISA 61).

**Fig 1:**
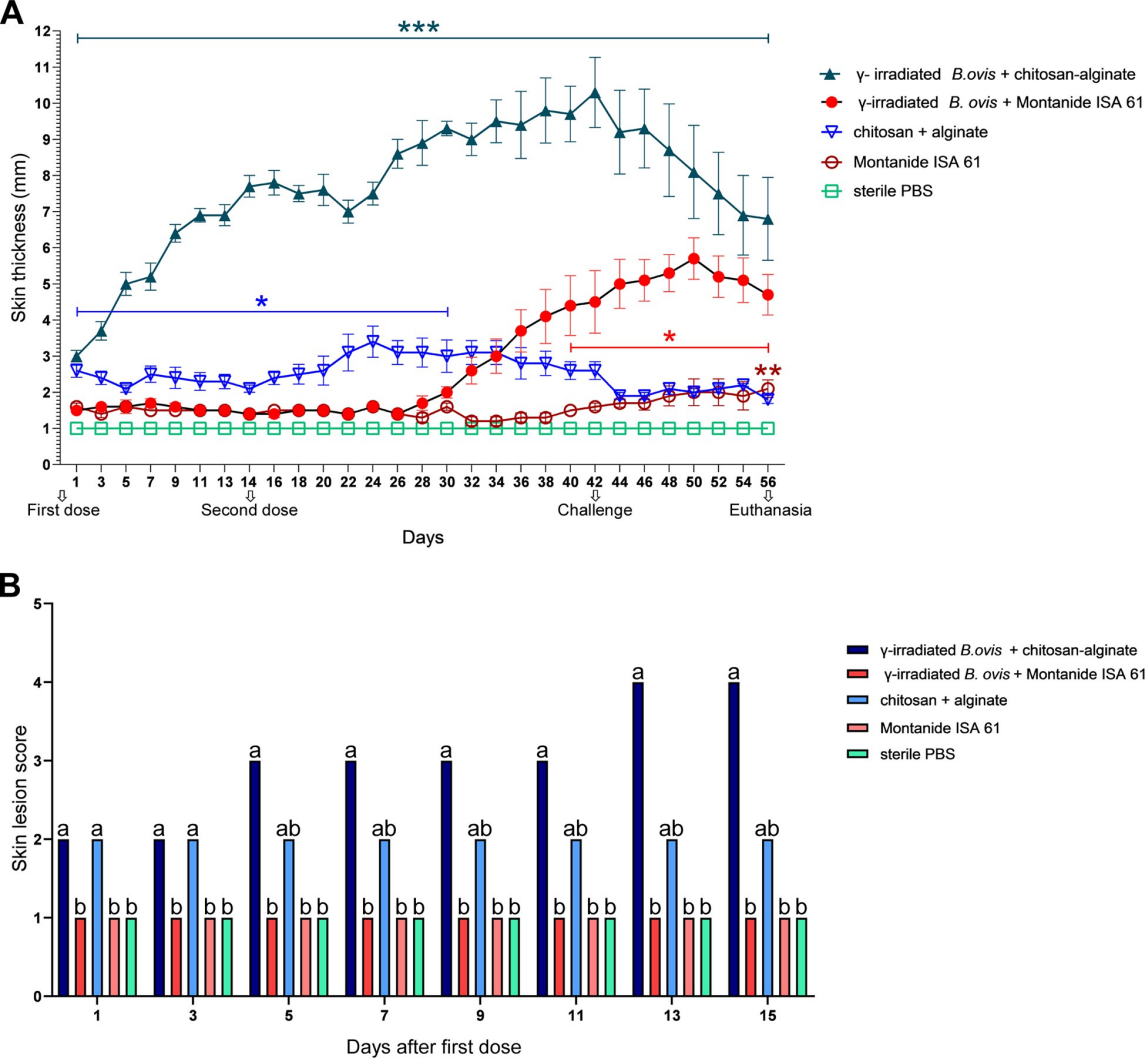
Changes at the inoculation site. (A) Skin thickness at the inoculation site of female C57BL/6 mice (n = 5) was evaluated for 56 days. Mice were subcutaneously inoculated with γ-irradiated *Brucella ovis* + alginate/chitosan, γ-irradiated *B*. *ovis* + Montanide ISA 61, chitosan-alginate, Montanide ISA 61, or sterile PBS (non-immunized). Data points represent mean skin thickness per group at the site of application. *** p < 0.001 (dark blue indicates significant differences between γ-irradiated *Brucella ovis* + alginate/chitosan and controls); ** p < 0.01 (brown indicates significant differences between Montanide ISA 61 and controls); * p < 0.05 (blue indicates significant differences between chitosan-alginate and controls; red indicates significant differences between γ-irradiated *B*. *ovis* + Montanide ISA 61 and controls). (B) Skin lesion scores at the inoculation site of female C57BL/6 mice (n = 5) after inoculation of first dose. Data represent the median of each group. Data were analyzed using the Kruskal-Wallis non-parametric test followed by Dunn’s multiple comparisons. Different letters indicate statistically significant differences at a given time point (p < 0.05).

Mice inoculated with γ-irradiated *B*. *ovis* + alginate/chitosan demonstrated a progressive and long-lasting increase in skin thickness, whereas mice inoculated with γ-irradiated *B*. *ovis* + Montanide ISA 61 developed increased thickness of the skin only at later points in the study (Fig 1A). Importantly, three mice inoculated with γ-irradiated *B*. *ovis* + alginate/chitosan developed draining fistulas with a purulent exudate associated with areas of ulceration. These lesions were not observed in any of the other groups. Mice inoculated with sterile PBS did not have any increase in skin thickness throughout the study.

In average, the skin thickness at the site of inoculation with γ-irradiated *B*. *ovis* + alginate/chitosan was significantly higher than that of mice inoculated with γ-irradiated *B*. *ovis* + Montanide ISA 61 as well as groups inoculated with only alginate/chitosan, Montanide ISA 61 or sterile PBS (p < 0.001) (Fig 1A). Similarly, lesion scores were significantly higher in mice inoculated with γ-irradiated *B*. *ovis* + alginate/chitosan compared to the other groups at 13 and 15 days after the second dose (Fig 1B).

**Fig 2:**
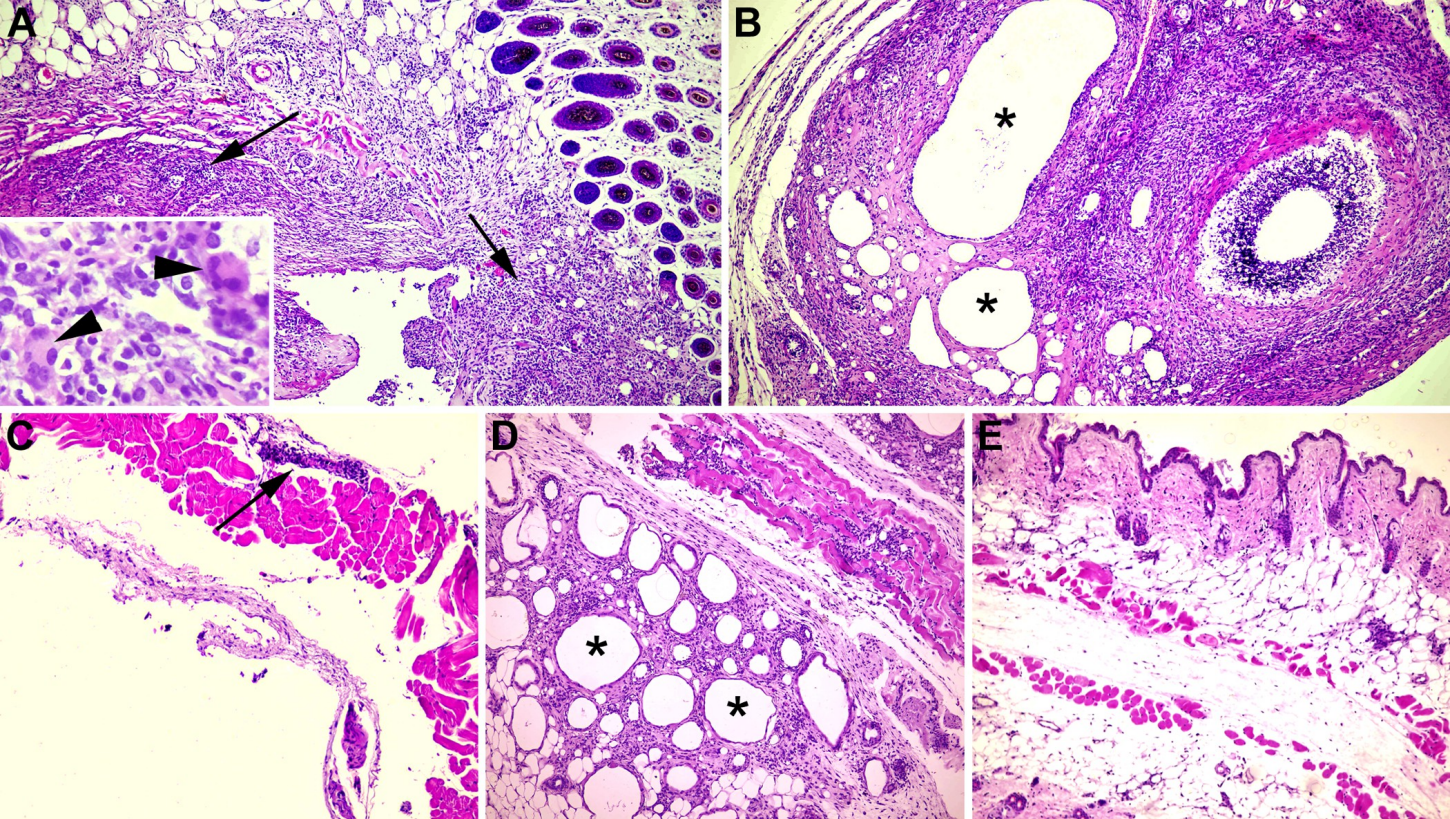
Lesions at the inoculation site of vaccine formulations in female C57BL/6 mice subcutaneously inoculated with γ-irradiated *Brucella ovis* + alginate/chitosan, γ-irradiated *B*. *ovis* + Montanide ISA 61, chitosan–alginate, Montanide ISA 61, or sterile PBS. (A) γ-irradiated *Brucella ovis* + alginate/chitosan: skin and subcutaneous tissue with dense infiltrate of plasma cells, lymphocytes and numerous neutrophils surrounded by foamy and epithelioid macrophages (arrows), occasionally forming multinucleated giant cells (inset and arrowheads) associated with an extracellular granular amphophilic material (interpreted as adjuvant). (B) γ-irradiated *B*. *ovis* + Montanide ISA 61: pyogranulomatous inflammation with well-defined white spaces of variable sizes consistent with the space left by the adjuvant (*), surrounded by variable numbers of degenerated and viable neutrophils, with aggregates of necrotic debris. (C) alginate/chitosan: mild lymphohistioplasmacytic infiltrate in the subcutaneous tissue (arrow). (D) Montanide ISA 61: pyogranulomatous panniculitis with numerous well-defined white spaces (*). (E) Sterile PBS: no significant histological changes. Hematoxylin and eosin, 10x objective.

Histologically, at the site of inoculation at 8 weeks after the first vaccination there was an intense inflammatory infiltrate composed of numerous epithelioid macrophages, neutrophils, lymphocytes and plasma cells was observed surrounded by abundant deposition of collagen fibers associated with fibrin, edema and cellular debris (Fig 2). There were moderate and multifocal amounts of granular material amphophilic or well-defined empty spaces (interpreted as remnants of adjuvants). The inflammation extended into the deep dermis, subcutaneous tissue and musculature. There were muscle fibers with loss of striations and hypereosinophilia (myocyte necrosis). These changes were compatible with a local reaction to the formulation; especially in groups containing the antigen (Fig 2A and 2B), since for the groups inoculated only with the adjuvants alginate/chitosan and Montanide ISA 61 the reaction was less pronounced (Fig 2C and 2D). Mice inoculated with sterile PBS had no significant histological changes at the inoculation site (Fig 2E).

None of the experimental groups lost weight and there was no significant differences in weight gain among all groups throughout the course of the study (S1 Fig).

### Immunization with γ-irradiated *Brucella ovis* encapsulated in alginate/chitosan or γ-irradiated *B*. *ovis* associated with Montanide ISA 61 induced a strong humoral immune response in mice challenged with wild-type *B*. *ovis*

**Fig 3:**
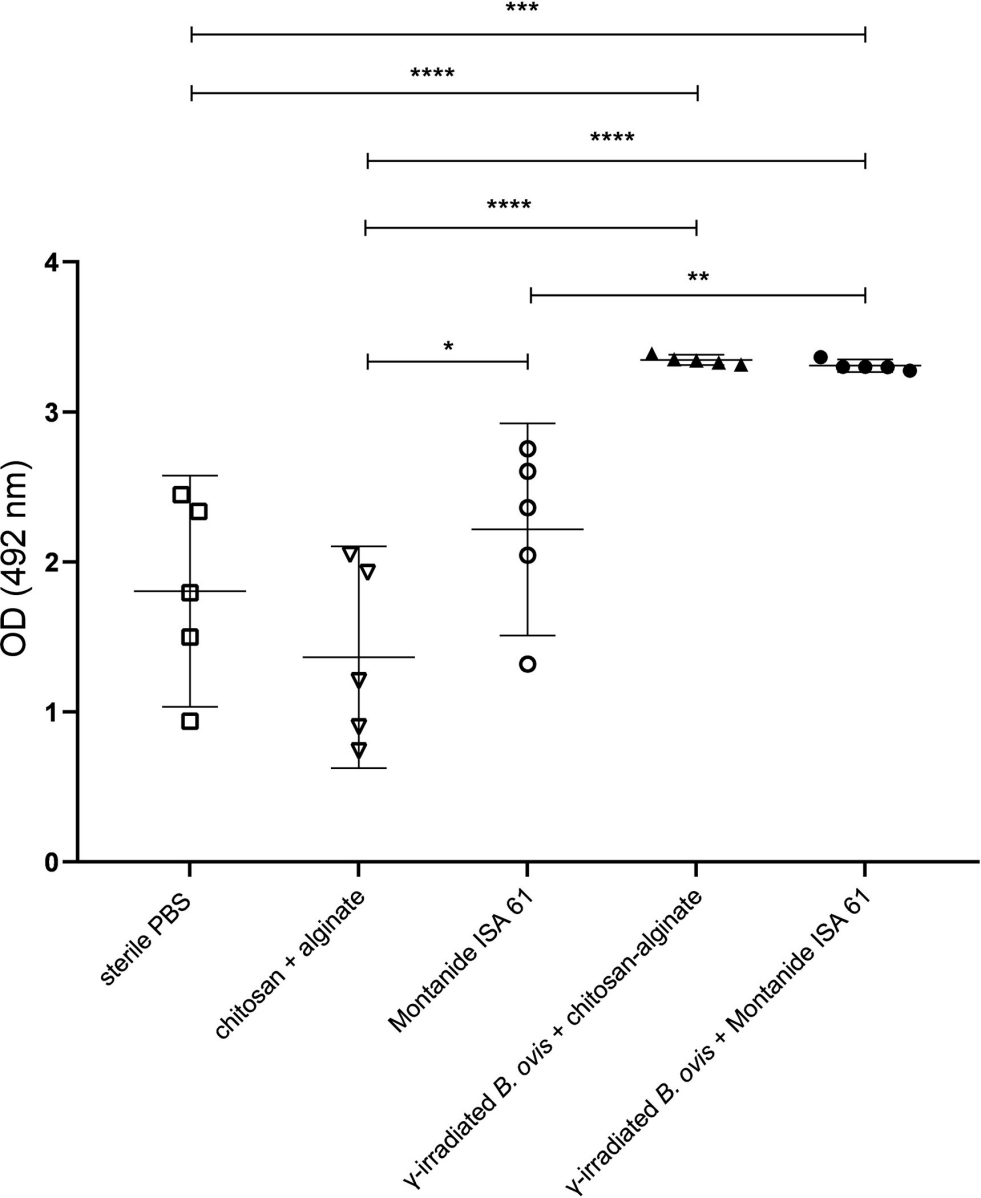
Anti-*Brucella ovis* IgG levels measured by ELISAi in sera from female C57BL/6 mice (n = 5) subcutaneously inoculated with γ-irradiated *B*. *ovis* + alginate/chitosan, γ-irradiated *B*. *ovis* + Montanide ISA 61, alginate/chitosan, Montanide ISA 61, or sterile PBS (non-immunized), and subsequently challenged with the wild type *B. ovis*. Data points represent individual mouse, means and standard deviation are indicated. Data passed normality test prior to ANOVA, and means were compared by Tukey’s test. Significant differences: * p < 0.05, ** p < 0.01, *** p < 0.001, and **** p < 0.0001.

**Fig 4:**
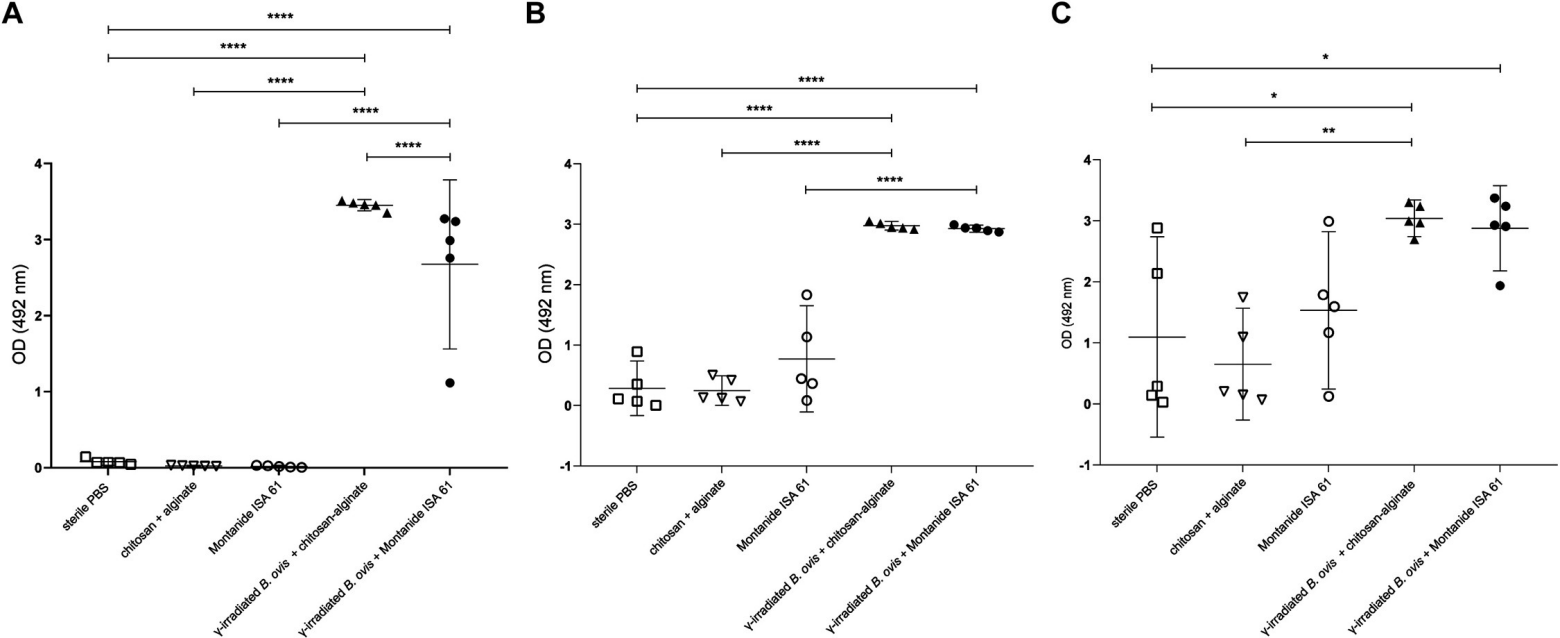
Levels of anti-*Brucella ovis* (A) IgG1, (B) IgG2b, and (C) IgG3 in sera from female C57BL/6 mice (n = 5) subcutaneously inoculated with γ-irradiated *B*. *ovis* + alginate/chitosan, γ-irradiated *B*. *ovis* + Montanide ISA 61, alginate/chitosan, Montanide ISA 61, or sterile PBS (non-immunized), and subsequently challenged with wild type *B*. *ovis*. Data points represent individual mouse, means and standard deviation are indicated. Data passed normality test prior to ANOVA, and means were compared by Tukey’s test. Significant differences: * p < 0.05, ** p < 0.01, *** p < 0.001, **** p < 0.0001.

Evaluation of IgG levels indicated that mice immunized with γ-irradiated *B*. *ovis* + alginate/chitosan or γ-irradiated *B*. *ovis* + Montanide ISA 61 and challenged with wild type *B*. *ovis* had significantly higher levels of total IgG when compared to the non-immunized mice or those that received only adjuvants (Fig 3). Immunoglobulin subclasses, namely IgG1, IgG2b and IgG3, were measured to assess Th1 or Th2 responses. A significant increase in the levels of IgG1, IgG2b and IgG3 subclasses was observed in mice immunized with γ-irradiated *B*. *ovis* + alginate/chitosan and γ-irradiated *B*. *ovis* + Montanide ISA 61. There were significantly higher levels of IgG1 induced by γ-irradiated *B*. *ovis* + alginate/chitosan when compared to γ-irradiated *B*. *ovis* + Montanide ISA 61 (Fig 4).

In regard to IgG2 subclasses, IgG2b was chosen since it is considered an indicator of a Th1 response in the C57BL/6 mouse strain [18, 19]. Considering the IgG2b/IgG1 ratio, γ-irradiated *B*. *ovis* + alginate/chitosan induced a ratio of 0.86 (IgG2b/IgG1 < 1). In contrast, γ-irradiated *B*. *ovis* + Montanide ISA 61 induced a ratio of 1.09, which indicates a predominantly Th1 profile (IgG2b/IgG1 > 1).

**Fig 5:**
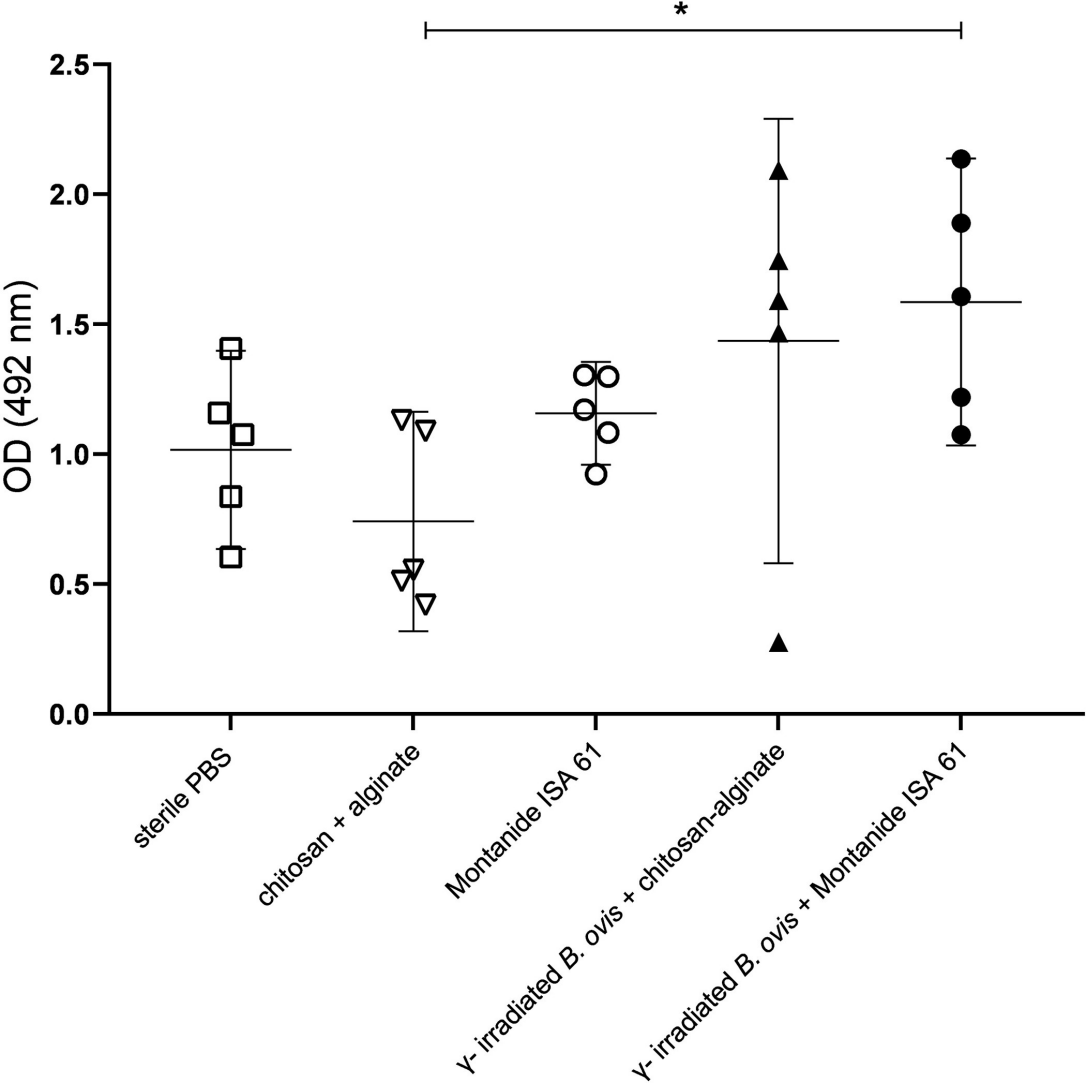
Levels of anti-*Brucella ovis* IgM in sera from female C57BL/6 mice (n = 5) subcutaneously inoculated with γ-irradiated *B*. *ovis* + alginate/chitosan, γ-irradiated *B*. *ovis* + Montanide ISA 61, alginate/chitosan, Montanide ISA 61, or sterile PBS (non-immunized), and subsequently challenged with wild type *B. ovis*. Data points represent individual mouse, means and standard deviation are indicated. Data passed normality test prior to ANOVA, and means were compared by Tukey’s test. Significant differences: * p < 0.05.

There was a significant difference in IgM levels between vaccinated groups and groups that received only the adjuvants or sterile PBS (Fig 5).

### Protection provided by vaccination with *Brucella ovis* γ-irradiated encapsulated with alginate/chitosan and *B*. *ovis* γ-irradiated associated with Montanide ISA 61

**Fig 6:**
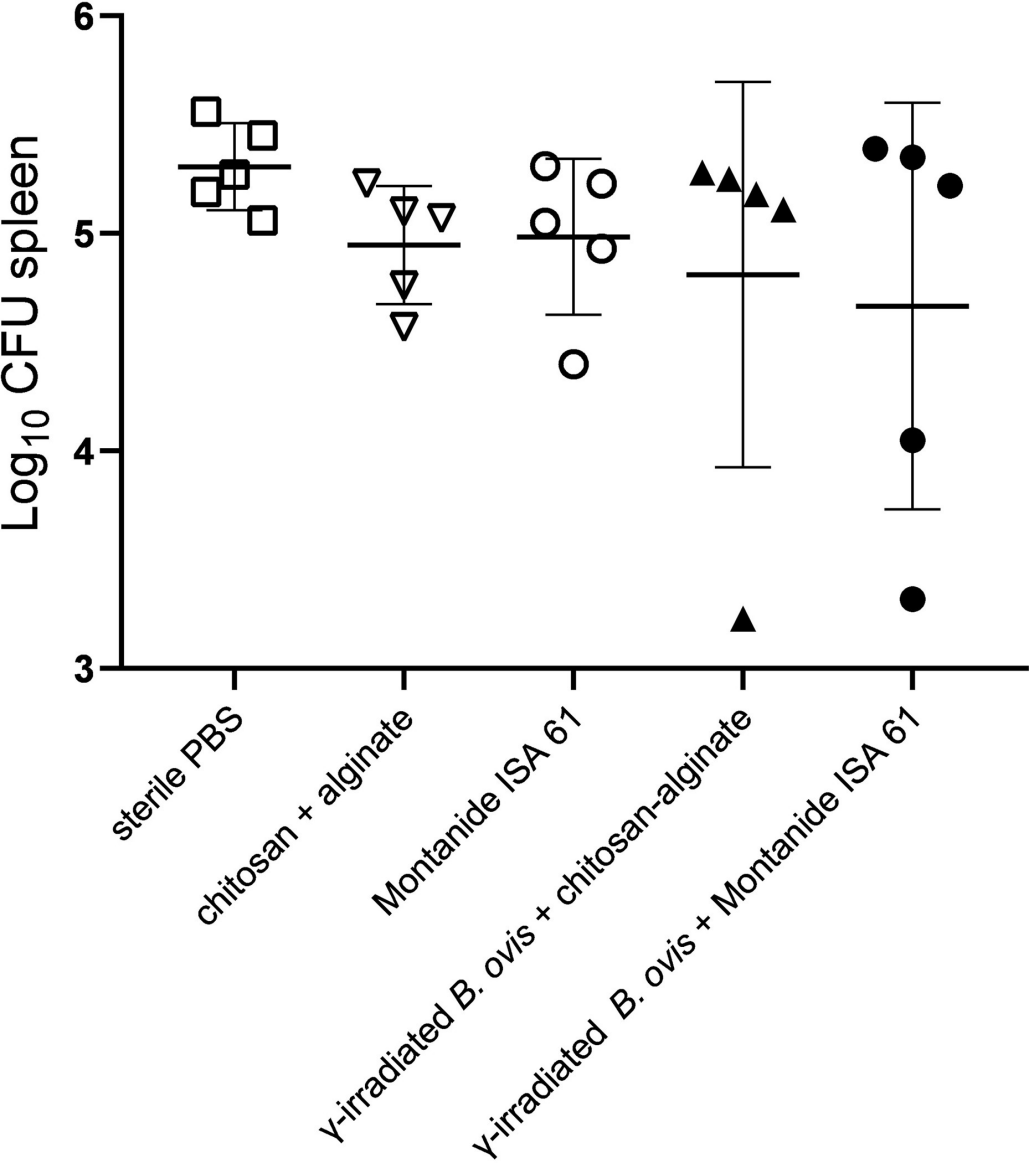
*Brucella ovis* CFU numbers in the spleen of female C57BL/6 mice (n = 5) subcutaneously inoculated with γ-irradiated *B*. *ovis* + alginate/chitosan, γ-irradiated *B*. *ovis* + Montanide ISA 61, alginate/chitosan, Montanide ISA 61, or sterile PBS (non-immunized), and subsequently challenged with wild type *B. ovis*. Data points represent individual mouse, means and standard deviation are indicated.

Among experimentally vaccinated animals, 1/5 and 2/5 mice had reductions in bacterial loads in the spleen that were equal or greater than 1 1og_10_ CFU in comparison to unvaccinated controls, when vaccinated with γ-irradiated *B*. *ovis* + alginate/chitosan or γ-irradiated *B*. *ovis* + Montanide ISA 61, respectively. However, in average there were no significant differences in CFU in the spleen of all experimental groups (Fig 6).

**Fig 7:**
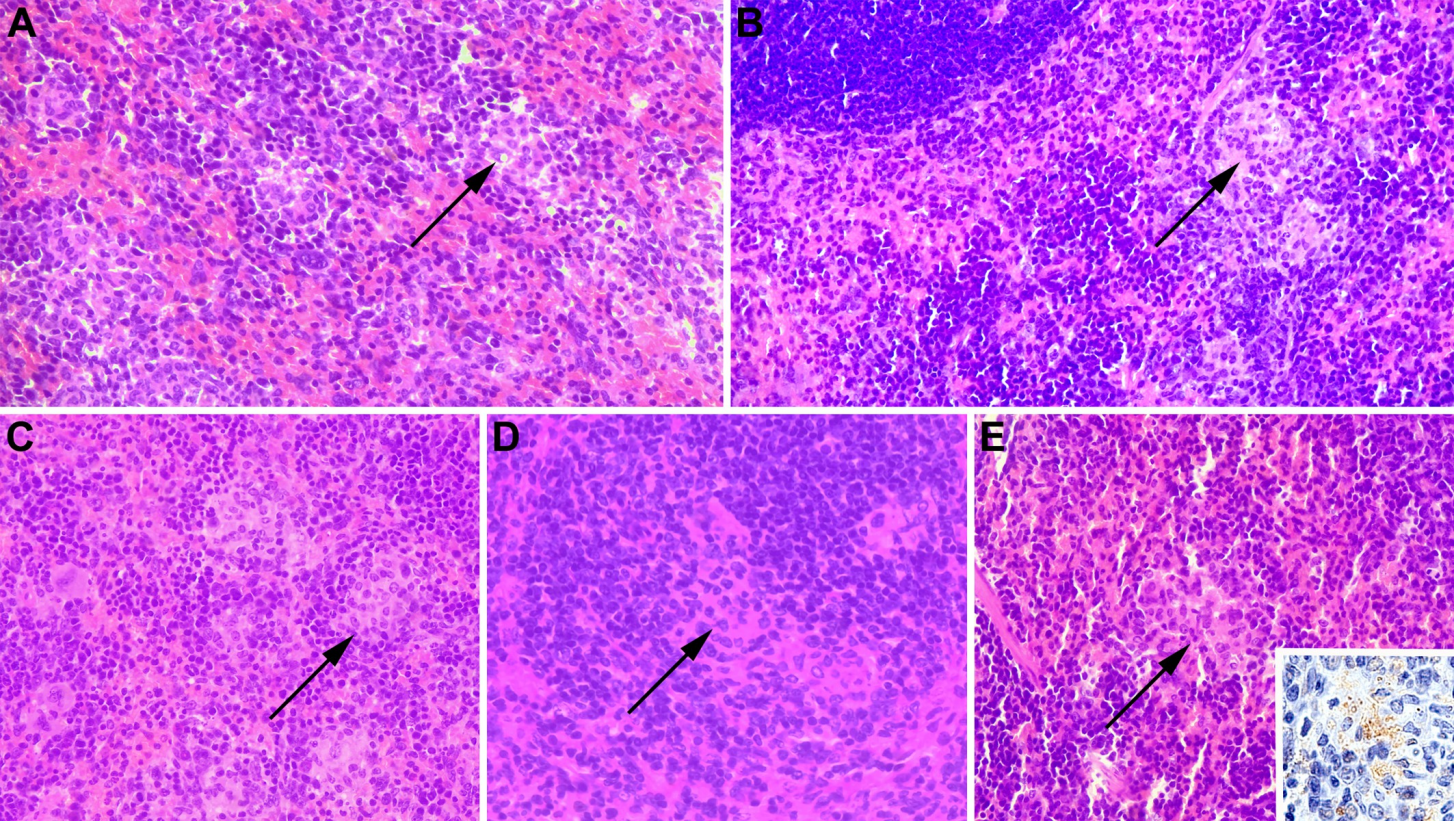
Spleen of female C57BL/6 mice subcutaneously inoculated with (A) γ-irradiated *Brucella ovis* + alginate/chitosan, (B) γ-irradiated *B*. *ovis* + Montanide ISA 61, (C) alginate/chitosan, (D) Montanide ISA 61, or (E) sterile PBS (non-immunized), subsequently challenged with wild type *B*. *ovis*. Microgranulomas (arrows) are observed in all groups, with larger foci of inflammatory cell accumulation in the groups that received only the adjuvants or non-immunized mice. H.E 40x. Inset in E: immunostaining of intralesional *Brucella* antigens.

Microscopically, all groups had multifocal areas of inflammatory cells, composed of epithelioid macrophages, lymphocytes and neutrophils associated with necrosis, compatible with microgranulomas in the spleen (Fig 7). Similarly, there were no significant differences in median histopathologic lesion scores among all groups (S2 Fig).

## Discussion

This is the first study that evaluated and compared the reactogenicity and the profile of immune response elicited by Montanide ISA 61 (emulsion) and chitosan coated alginate (biopolymers) associated with γ-irradiated *B*. *ovis* in a murine model. Reactogenicity represents the physical manifestation of reactions that occur after vaccination, including inflammatory or hypersensitivity responses and can include pain on the injection site, redness, swelling or induration at the injection site, or systemic symptoms. Adjuvants usually increase reactogenicity compared to inactivated vaccines or purified antigens without adjuvant [20]. Though chitosan as a biopolymer is generally considered biocompatible, biodegradable and thus has low reactogenicity, we observed intense local reactogenicity with skin ulcers and fistulas in the subcutaneous tissue of some mice submitted to immunization with chitosan coated alginate in this study, especially when associated with the antigens as indicated by the lesion scores (Fig 1B). Vasiliev (2015) [21] emphasizes that standardized parameters for chitosan such as molecular weight and viscosity (assessed by chromatography and viscosimetry), deacetylation degree (nuclear magnetic resonance) and endotoxin level (limulus amebocyte lysate test), are important for reliable preclinical and clinical studies. In this context, purity is an important issue since the biological origin of chitosan could determine its contamination with endotoxins, LPS and other substances that have intrinsic immune modulating activities [21]. In this study, a commercially available chitosan was employed and some of these parameters were properly monitored (as described in the material and methods section).

Adverse reactions at the site of inoculation that cause pain or tissue damage are the main obstacles for development and licensing of vaccines containing adjuvants with immunostimulatory activity. The results of this study demonstrated by the score of lesions that Montanide ISA 61 has less reactogenicity, when compared to alginate/chitosan biopolymers and corroborate with Petermann et al. (2017) [22] who observed increased skin thickness as the only adverse effect to vaccination with Montanide adjuvant in cattle. Despite the low reactogenicity, Montanide ISA 61 promoted an intense and long-lasting local inflammatory response as demonstrated by histopathological findings (Fig 2B).

Development of the appropriate type of immune response is essential for protection. In mice, Th1 or Th2 responses are associated with expression of specific cytokines, such as IFN-γ (a Th1 profile cytokine related to the production of IgG2a, IgG2b and IgG3), and IL4 (a Th2 profile cytokine related to IgG1 production). Th1 profile associated with IFNγ synthesis plays an important role for protective immunity against *Brucella* [23, 24].

In this study, Montanide ISA 61 associated with γ-irradiated *B*. *ovis* and alginate/chitosan with γ-irradiated *B*. *ovis* induced high levels of IgG1, IgG3 and IgG2b. IgG2b is an important Th1 response marker in C57BL/6 mice [19]. IgG3 is associated with protection against various infectious diseases [25] and it has been previously investigated as an indication of immune response against *Brucella* [26]. Immunoglobulin levels in these groups had significant differences in comparison to groups that received only the adjuvants and to non-immunized animals. However, when evaluating the IgG2b/IgG1 ratio, it was observed that only the formulation γ-irradiated *B*. *ovis* + Montanide ISA 61 resulted in a value greater than 1, suggesting a predominance of Th1 immune response. Despite significant differences in IgM levels between vaccinated groups and groups that received only the adjuvants or sterile PBS, IgM levels were likely influenced not only by vaccination but also by challenge with wild type *B*. *ovis*, which was performed two weeks before euthanasia. Indeed, IgM usually is detected early during infection, while specific IgG antibodies tend to develop later but often remain detectable for long periods of time [27].

In the mouse model of brucellosis, reduction in bacterial loads in the spleen of vaccinated mice compared to unvaccinated mice is commonly used to demonstrate a protective action of experimental vaccines [15, 26] In this study, the inactivated experimental vaccine formulations based on alginate/chitosan or Montanide ISA 61, resulted in bacterial loads < 1 1og_10_ CFU in comparison to unvaccinated controls in 1/5 and 2/5 mice, respectively. This limited experimental vaccine response may be due to the broadly accepted notion that live attenuated vaccines tend to be more protective against intracellular bacteria [28]. There were no significant differences in the histopathology scores of spleen, which is in good agreement with many experiments in brucellosis vaccinology employing the mouse model, in which bacterial loads in the spleen is a better surrogate of protection that histopathologic scores [15].

In conclusion, the emulsion Montanide ISA 61 as well as the biopolymers alginate/chitosan elicited a marked and lasting local response, especially when associated with the antigen. However, Montanide resulted in milder reactogenicity when compared to the alginate/chitosan. In addition, Montanide ISA 61 associated with γ-irradiated *B*. *ovis* induced high levels of IgG2b and demonstrated by IgG2b/IgG1 ratio, a Th1 profile response. Thus, the Montanide ISA 61 emulsion may be a viable alternative for future studies involving other intracellular bacteria and vaccine formulations.

## Supporting information

S1 Table Raw data generated in this study. (XLSX)

S1 Fig Weight gain of female C57BL/6 mice (n = 5) inoculated with γ-irradiated *B*. *ovis* + alginate/chitosan, γ-irradiated *B*. *ovis* + Montanide ISA 61, chitosan-alginate, Montanide ISA 61, or sterile PBS (non-immunized) during the course of the experiment (56 days). No statistical difference was observed between the groups. The results were analyzed for normality before being submitted to ANOVA, with the mean values compared by Tukey’s test (p > 0.05).
(TIF)

S2 Fig Histopathological score. Medians group were analyzed using the non-parametric Kruskal-Wallis test. There was no significant difference between groups (p > 0.05).
(TIF)
