# Supplementary figures and images for: Comparative study on alginate/chitosan microcapsules and Montanide ISA 61 as vaccine adjuvants in mice
Source: PLoS One. 2024 Apr 4;19(4):e0298117. doi: 10.1371/journal.pone.0298117 (PMC10994407; doi:10.1371/journal.pone.0298117)

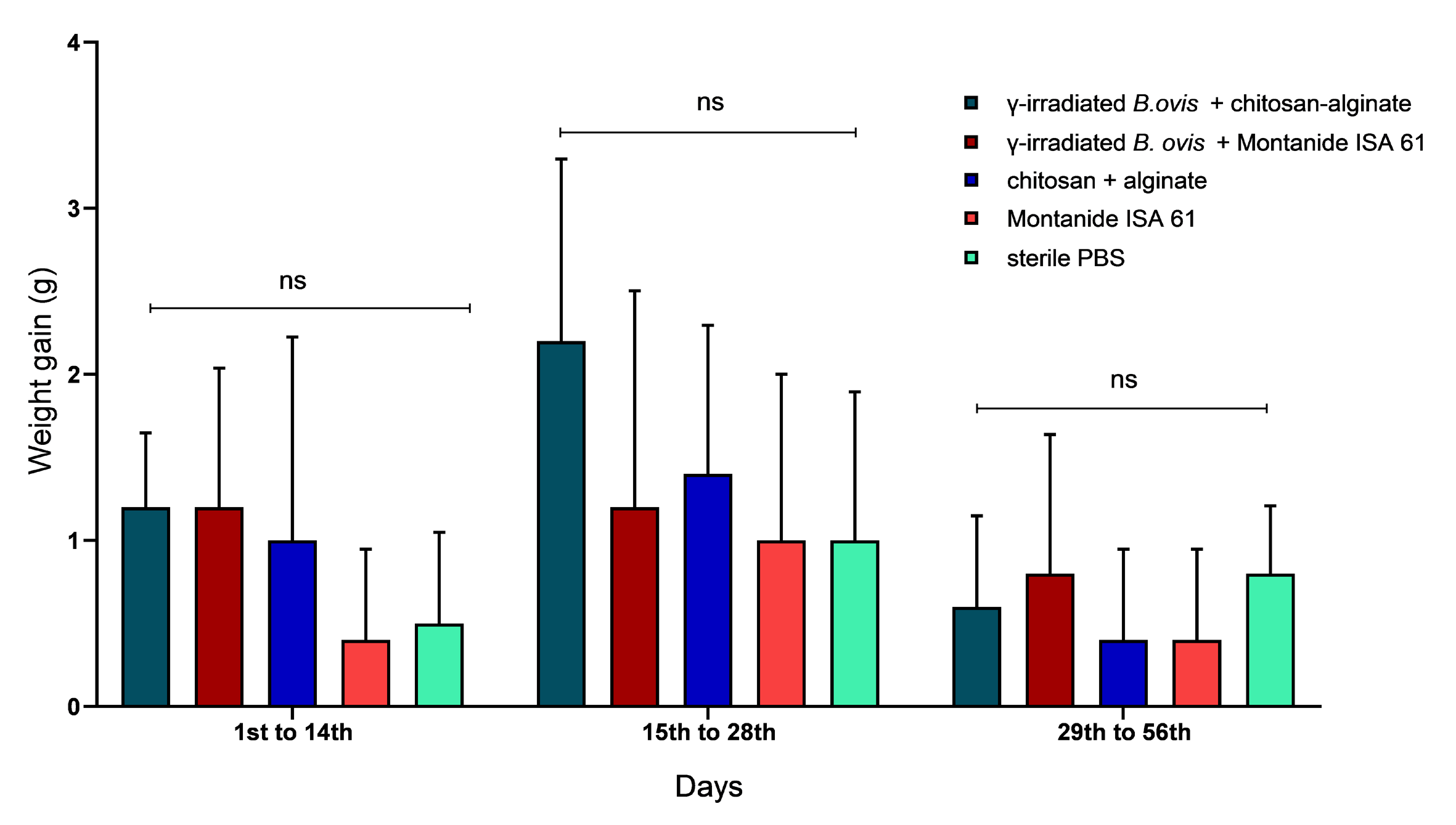

Supplement: S1 Fig — No statistical difference was observed between the groups. The results were analyzed for normality before being submitted to ANOVA, with the mean values compared by Tukey’s test (p > 0.05). (TIF) [file pone.0298117.s002.tif]

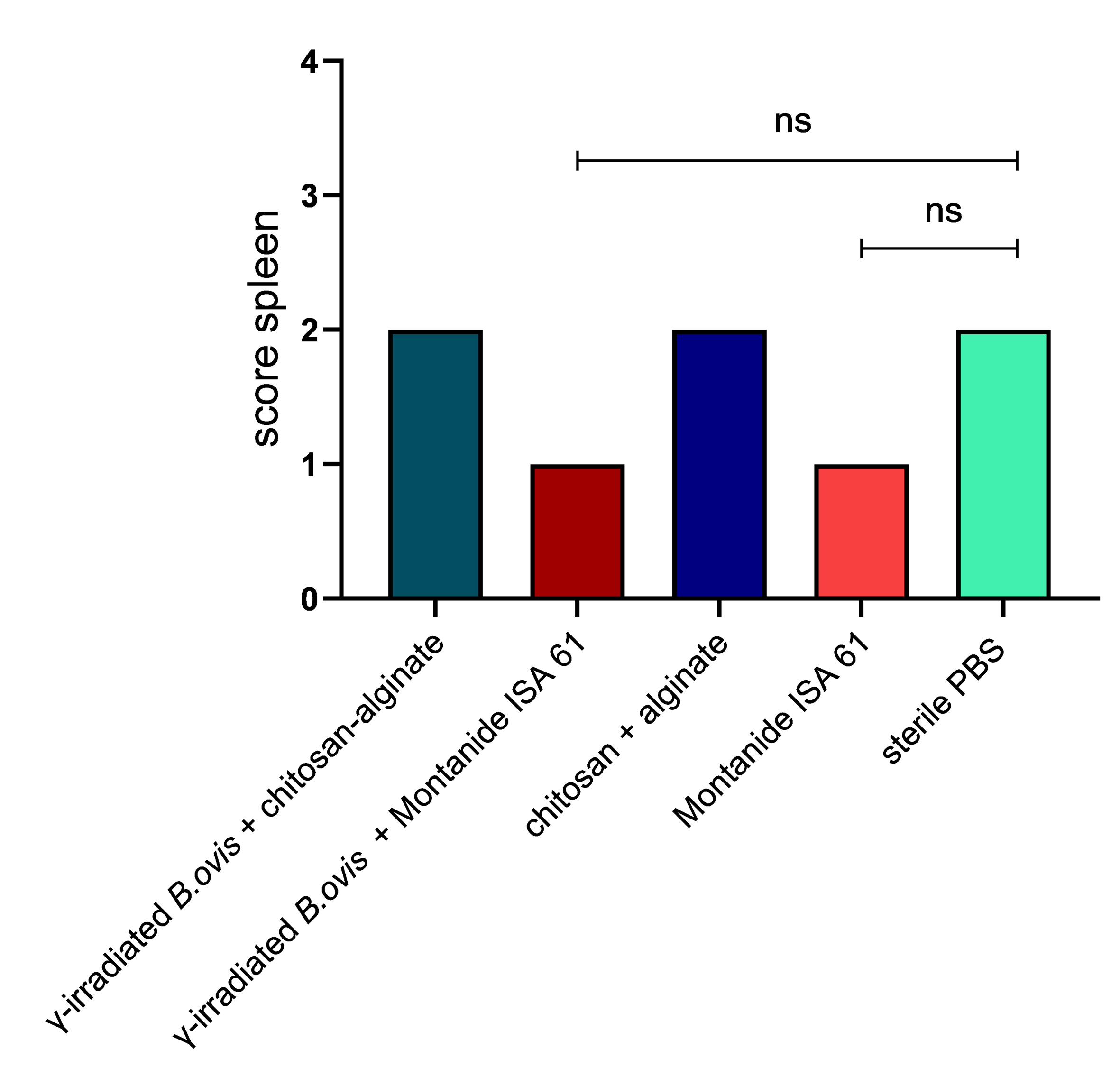

Supplement: S2 Fig — Medians group were analyzed using the non-parametric Kruskal-Wallis test. There was no significant difference between groups (p > 0.05). (TIF) [file pone.0298117.s003.tif]
